# The Conceivable Functions of Protein Ubiquitination and Deubiquitination in Reproduction

**DOI:** 10.3389/fphys.2022.886261

**Published:** 2022-07-13

**Authors:** Jiayu Wang, Qi Zhou, Jinli Ding, Tailang Yin, Peng Ye, Yan Zhang

**Affiliations:** ^1^ Reproductive Medicine Center, Renmin Hospital of Wuhan University, Wuhan, China; ^2^ Hubei Clinic Research Center for Assisted Reproductive Technology and Embryonic Development, Wuhan, China; ^3^ Department of Pharmacy, Renmin Hospital of Wuhan University, Wuhan, China; ^4^ Department of Clinical Laboratory, Renmin Hospital of Wuhan University, Wuhan, China

**Keywords:** ubiquitin, deubiquitinating enzymes, early embryonic development, gametogenesis, fertilization

## Abstract

Protein ubiquitination with general existence in virtually all eukaryotic cells serves as a significant post-translational modification of cellular proteins, which leads to the degradation of proteins via the ubiquitin–proteasome system. Deubiquitinating enzymes (DUBs) can reverse the ubiquitination effect by removing the ubiquitin chain from the target protein. Together, these two processes participate in regulating protein stability, function, and localization, thus modulating cell cycle, DNA repair, autophagy, and transcription regulation. Accumulating evidence indicates that the ubiquitination/deubiquitination system regulates reproductive processes, including the cell cycle, oocyte maturation, oocyte-sperm binding, and early embryonic development, primarily by regulating protein stability. This review summarizes the extensive research concerning the role of ubiquitin and DUBs in gametogenesis and early embryonic development, which helps us to understand human pregnancy further.

## Introduction

Back in 1975, Goldstein et al. (1975) and Schlesinger and Goldstein (1975) first discovered a free protein that was ubiquitous in mammalian cells, yeast, bacteria, and higher plants. For this reason, the protein was named ubiquitin. Ubiquitin is an 8.6 kDa peptide composed of 76 amino acids. The structure of ubiquitin is roughly a compact sphere with an unrestrained and flexible C-terminal tail (Ramage et al., 1994). The first ubiquitin molecule is covalently bound through its C-terminal carboxylate group to the target protein. Polyubiquitylation occurs when the C-terminus of another ubiquitin is linked to the lysine residues or N-terminus of the first ubiquitin. There are seven lysine residues (K6, K11, K27, K29, K33, K48, and K63) and the N-terminus (M1) on ubiquitin, which are all potential sites for polyubiquitination (Callis, 2014). Ubiquitin is highly conserved and present in many organisms, which gives ubiquitination an identical meaning across the vast evolutionary distance from yeast to humans (Oh et al., 2018). The genome of an archeal species, *Caldiarchaeum subterraneum*, was sequenced, and the existence of a full set of ubiquitin was found by Nunoura et al. (2011). Ubiquitin has remained virtually unchanged for billions of years, during which it has performed unique biological functions in a heterogeneous and ever-changing cellular environment. Furthermore, ubiquitin tolerates a wide range of pH and temperatures and is highly resistant to trypsin digestion (Vijay-Kumar et al., 1985). The highly conserved and highly stable nature of ubiquitin makes the study of the biological functions and molecular mechanisms of ubiquitin a hot research topic. These properties also provide us with many facilities to study the roles of ubiquitin.

Ubiquitin labeling determines the fate of proteins. With a wide range of possible patterns and reversibility, ubiquitination can take many shapes to meet the specific needs of a given cell in time and space. The ubiquitination process regulation is mainly accomplished by the binding of E3 ligases to particular targets. The ubiquitination/deubiquitination system plays an essential role in protein degradation, cell cycle progression, and transcriptional regulation. Protein ubiquitination and deubiquitination play important roles in the key events in gametogenesis and early embryo development, including meiosis, sperm capacitation, and maternal-to-zygotic transition (MZT). Understanding the molecular mechanisms related to ubiquitination during embryogenesis and early embryonic development provides new clinical diagnosis and treatment targets.

## Study of Ubiquitination

### Enzymes Required for Ubiquitination

Ubiquitination is a PTM process involving covalent conjugation of ubiquitin to the substrate proteins (Swatek and Komander, 2016). The binding sites in substrate proteins include lysine (most typical), cysteine, serine, threonine residue, and N-terminus. As mentioned above, three sequentially activated enzymes drive the ubiquitination process: ubiquitin-activating enzymes (E1s), ubiquitin-conjugating enzymes (E2s), and ubiquitin ligases (E3s). These three enzymes accomplished the activation, binding, and ligation of ubiquitin.

During the initial ubiquitination step, E1s activates ubiquitin in an ATP-dependent manner, forming a thioester bond between the active-site cysteine sulfhydryl group of E1s and the C-terminal glycine of ubiquitin. Then, activated ubiquitin is transferred onto the catalytic cysteine residue E2s in the presence of ATP, which contributes to determining the type of substrate ubiquitination. Finally, under the coordination of a specific E3 that determines substrate specificity, ubiquitin is covalently bound to a substrate (Zheng and Shabek, 2017). The E3 enzymes interact with E2 enzymes to transfer ubiquitin molecules attached to E2-bound ubiquitin molecules to their target proteins. As the ubiquitin molecule attaches to the substrate protein, it catalyzes the covalent bond between the lysine residue inside the substrate and the C-terminus of ubiquitin. Therefore, E3-type ligases function as substrate-specific components of ubiquitination, determining the type of proteins to be ubiquitinated.

### Function of Ubiquitination

The proteins can be conjugated with monoubiquitin, multi-monoubiquitin, or a chain of ubiquitin (Sadowski et al., 2012). The basis of polyubiquitin chain formation is monoubiquitin, which means that after the first ubiquitin binds to the substrate protein, the glycine residue of the free ubiquitin is attached to the ubiquitination site (K6, K11, K27, K29, K33, K48, K63, and M1) of the first ubiquitin as mentioned above. The ubiquitin chain can be either homogeneous or heterogeneous depending on its internal linkages (Kirkpatrick et al., 2006; Dimova et al., 2012). The naming and classification of polyubiquitin chains are according to the binding sites. Monoubiquitination serves multiple cellular roles, while polyubiquitination was initially assumed to identify proteins for degradation (Chau et al., 1989). As the research continued, it was found that different types of polyubiquitin chains have different effects on the target protein.

The K48-linked polyubiquitin chains function as targets for the degradation of proteins via the 26S proteasomal and lysosomal targeting signals (Komander and Rape, 2012). The proteasome degrades ubiquitin-tagged proteins by breaking their peptide bonds. The 26S proteasome consists of a 20s subunit and two 19s regulatory cap subunits. The cap structure recognizes K48-polyubiquitinated proteins and transfers them to the catalytic core 20s subunit within the proteasome (Dong et al., 2019). Instead of disrupting the ubiquitin, the proteasome recycles them back into the free ubiquitin pool (Collins and Goldberg, 2017). It has recently been demonstrated that the signals for proteasomal targeting also included K6, K11, K27, K29 M1, and the heterogeneous ubiquitin chains.

However, K63 polyubiquitinated proteins are selectively bound by the ESCRT0 to block the recognition of the proteasome (Nathan et al., 2013). Therefore, K63-linked polyubiquitin chains play various roles in non-degradative signaling processes or can also target the proteins for degradation by lysosomes (Jackson and Durocher, 2013).

The ubiquitination system degrades the regulatory proteins and damaged or misfolded polypeptides, which allows ubiquitylation to function as a modulator for maintaining intracellular amino acid and protein homeostasis (Collins and Goldberg, 2017). It also has another consequential output as the regulator of cell signaling network plus the cell cycle by regulating protein interactions and activities, resembling phosphorylation and other PTM (Walczak et al., 2012; Swatek and Komander, 2016). The ubiquitination system regulates diverse indispensable cellular processes, including DNA repair, cell cycle, autophagy, transcription regulation, etc.

### Writer, Reader, and Eraser

**FIGURE 1:**
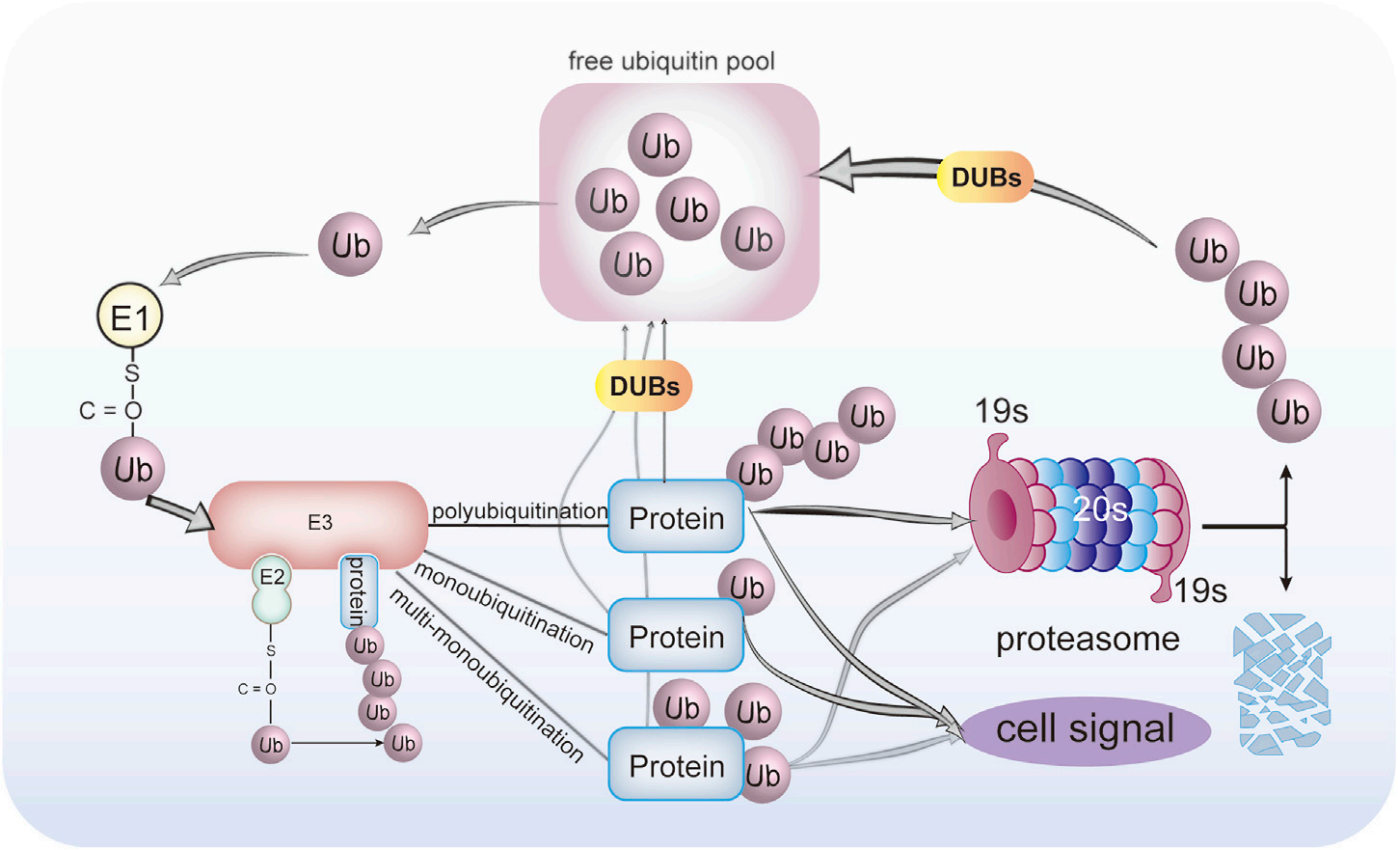
Protein ubiquitination and deubiquitination process. Ubiquitin was activated by E1 ubiquitin-activating enzyme in an ATP-dependent manner to form a thioester bond between the active-site cysteine sulfhydryl group of the E1 enzyme and the C-terminal glycine of ubiquitin. Then, activated ubiquitin is transferred onto the catalytic cysteine residue E2 ubiquitin-conjugating enzyme in the presence of ATP, which determines the type of substrate ubiquitination. Finally, under coordination by a specific E3 ligase that determines substrate specificity, ubiquitin is covalently bound to a substrate. The proteins can be conjugated with either monoubiquitin, multi-monoubiquitin, or polyubiquitination. The DUBs can reverse this process. The ubiquitinated proteins can either be degraded by the 26S proteasome or transduced cell signal. The DUBs can remove and recycle the ubiquitin chains to regenerate free ubiquitin.

The ubiquitination proceeds, along with many other post-translational modifications (PTM), in a tightly regulated manner, comparable to the “writer, reader, and eraser.” The “writer” of the ubiquitin signals is the E1-E2-E3 enzymatic cascade. These signals are “read” by ubiquitin-binding domains (UBDs) or ubiquitin receptors on the proteasome or downstream signaling pathway which contain many UBDs (Dikic et al., 2009). The UBDs bind non-covalently to ubiquitin (Hicke et al., 2005). The UBDs are widely distributed in intracellular proteins, such as the 19s cap subunit of the proteasome, signaling molecules regulated by ubiquitination, and some deubiquitinating enzymes (deubiquitylases or deubiquitinases; hereafter DUBs). DUBs can “erase” the ubiquitination modifications of the substrate protein, as in Figure 1. The DUBs can reverse the ubiquitinated protein; that is to say, DUBs remove ubiquitin from the substrate proteins. This makes the ubiquitination process precisely regulated and reversible. The human genome encodes more than 90 family members of DUBs (Nijman et al., 2005). These DUBs are grouped into six subfamilies, ubiquitin-specific proteases (USPs), ovarian tumor proteases (OTUs), ubiquitin carboxyl-terminal hydrolases (UCHs), Josephin family, JAMM/MPN domain-associated metallopeptidases (JAMMs), and monocyte chemotactic protein-induced proteins (MCPIPs). Five DUBs subfamilies are cysteine proteases, and only JAMMs are metalloproteases.

### Function of DUBs

Functionally, DUBs belong to the following categories:

i). DUBs participate in the cleavage of free ubiquitin from translational proproteins. Ubiquitin is usually fused with ribosomal proteins or expressed as a linear fusion of multiple monoubiquitins. The polyubiquitin gene product has a supplementary small C-terminal adduct that calls for hydrolysis by DUBs to generate (Kimura et al., 2009).

ii). DUBs are highly involved in the removal of ubiquitin or ubiquitin-like proteins from target protein conjugates, resulting in the reversal of ubiquitin signaling, and therefore, stabilizing proteins by rescue from their fate of degradation (Tsuchiya et al., 2018). If the target protein is doomed to degradation anyway, DUBs can remove as well as recycle the ubiquitin chains before degradation by the proteasome (Kim et al., 2003). The unanchored polyubiquitin released from the substrate protein requires DUBs to regenerate free ubiquitin. The DUBs play a critical role in maintaining free ubiquitin homeostasis in the cell (Park and Ryu, 2014). In the meantime, this also implicates the involvement of DUBs in ribosome biogenesis (Hanna et al., 2007; Woo et al., 2019).

iii). DUBs can alter the Ub signal to modify the mode of ubiquitination and deubiquitination by trimming the ubiquitin chains. The dynamic balance between ubiquitination and deubiquitination is crucial for maintaining cellular proteostasis and regulating the protein activity (Leznicki and Kulathu, 2017).

By regulating ubiquitination, DUBs control various cell functions, such as proteasome- and lysosome-dependent proteolysis, progression of the cell cycle, gene expression, chromosomal segregation, kinase activation, localization, apoptosis, DNA repair, stemness maintenance, degradation of signaling intermediates, and spermatogenesis. The point of this review is how the universal language of ubiquitin modification plays a role in gametogenesis during the reproductive process.

## Functions of Protein Ubiquitination and Deubiquitination in Sperm

### Protein Ubiquitination and Deubiquitination in Spermatogenesis and Pre-Ejaculatory Maturation

Spermatogenesis goes through three stages of spermatogonial stem cell differentiation, spermatocyte meiosis, and sperm cell deformation, and finally develops into mature sperm. Mitosis allows spermatogonial stem cell (SSC) self-renewal as well as differentiation to maintain exquisite stem cell homeostasis (de Rooij, 2001). Infantile gonocytes differentiate into SSCs as the individual grows and develops. The E1s UBA6 appears to be involved in the regulation of mitosis. The cytoplasmic-to-nuclear transition of UBA6 is associated with the functional changes as the gonocytes differentiate (Hogarth et al., 2011). Additionally, the E2s Ube2e3 is more abundant in spermatogonia than in gonocytes and has been reported to prevent cellular senescence in other cell types (Plafker et al., 2018) see Table 1. The E3 ligase has continuously expressed in germ cells such as gonads, spermatogonia, and spermatocytes, of which the E3s Huwe1 maintains the characteristics of spermatogonia by regulating the expression of γ-H2AX and preventing the overactivation of the DNA damage (Bose et al., 2017). The balance of spermatogonial stem cell self-renewal and differentiation is disrupted in *Huwe1* knockout mice, which exhibited Sertoli cell-only syndrome. The specific inactivation of Huwe1 leads to the depletion of spermatogonia and the increase of spermatocytes in the pre-leptotene phase, destroying the establishment and maintenance of the spermatogonial stem cell pool, and unable to form a normal number of mature sperm, resulting in male infertility (Fok et al., 2017). Among DUBs, USP3 and UCHL1 are higher in germ cells Table 1. USP3 has been proved to regulate the DNA damage response through deubiquitination of its substrate ubiquitinated H2A and works as a chromatin modifier to maintain genome integrity in other human cells (Nicassio et al., 2007). To maintain the stem cell pool and differentiation of progeny, spermatogonia undergos both symmetrical and asymmetrical division. The asymmetric segregation of the UCHL1 protein between two daughter cells follows asymmetric division (Luo et al., 2009). UCHL1 maintains the competence of SSC differentiation in mice (Alpaugh et al., 2021). Accordingly, UCHL1 is a potential intrinsic determinant of differentiation or self-renewal of spermatogonia. The specific functions of these differentially expressed genes and their roles in human spermatocytogenesis and male infertility deserve continuous exploration.

**TABLE 1:** Ubiquitination and deubiquitination machinery and targeted substrates work to regulate sperm development and function.

Enzyme	Substrate	Ub chain	Function of ubiquitination		Role in sperm	Model		Reference
E1 enzyme								
UBA6					Gonocytes differentiate	Rat		Hogarth et al. (2011)
UBA1					Induce acrosomal exocytosis	*C. elegans* boar		Kulkarni and Smith, (2008) Yin et al. (2012)
E2 enzyme								
Ubc2e3					Rich in spermatogonia	Rat		Plafker et al. (2018)
HR6					Homologous recombination repair	Mouse		Roest et al. (1996)
E3 ligase								
HUWE1	Histone H2AX	Poly-ub		Pro deg	Initial differentiation of spermatogonia	Mouse		Bose et al. (2017) Fok et al. (2017)
UBR2	Histone H2A	Mono-ub		Transcriptional silencing	Homologous recombination repair	Mouse		Kwon et al. (2003)
RNF4	Checkpoint mediator MDC1	SUMO		MDC1 removal from chromatin	Repair of DSBs	Mouse		Galanty et al. (2012) Vyas et al. (2013)
RNF20	Histone H2B	Mono-ub		Recruitment of DSB repair factors	DSB repair chromatin remodeling	Mouse		Xu et al. (2016)
NEDL2	Unclear	unclear		Cellular function	DNA decondensation	Boar		Mao et al. (2020)
DUB								
USP3	Ubiquitinated H2A				DNA damage response		Human	Nicassio et al. (2007)
UCHL1	Unclear				SSC differentiation		Boar Mouse	Luo et al. (2009) Alpaugh et al. (2021)
ataxin-3	SUMOylated-MDC1				DSB repair		Human	Pfeiffer et al. (2017)
USP2	Unclear				Penetrating the ZP		Mouse	Bedard et al. (2011)
USP8	Membrane receptor MET				Endosomal sorting acrosome biogenesis		Mouse	Berruti and Paiardi, (2015) Berruti et al. (2010)

UBA6, ubiquitin-like modifier activating enzyme 6; UBA1, ubiquitin-like modifier activating enzyme 1; HR6, homolog of Rad6; HUWE1, HECT, UBA, and WWE domain containing E3 ubiquitin protein ligase 1; RNF20, ring finger protein 20; UBR2, ubiquitin protein ligase E3 component N-recognin 2; RNF4, ring finger protein 4; NEDL2, nedd4-related E3 ubiquitin ligase-2; USP3, ubiquitin-specific peptidase 3; UCHL1, ubiquitin C-terminal hydrolase L1; USP2, ubiquitin-specific peptidase 2; USP8, ubiquitin-specific peptidase 8; Pro deg, protein degradation; Mono-ub, monoubiquitin; Poly-ub, polyubiquitination; DSB double-strand break; SSC spermatogonial stem cell;

Meiosis causes the conversion of diploid spermatocytes into haploid spermatids. In this process, the homologous recombination produces genetic diversity and ensures the accuracy of chromosome transmission from generation to generation. Histone ubiquitination recruits and activates DNA double-strand break (DSBs) to repair proteins, and interacts with other PTMs such as histone methylation and acetylation to coordinately regulate the homologous chromosomal recombination (Swatek and Komander, 2016; Wang et al., 2018). HR6B is an essential E2 enzyme for spermatogenesis. *Hr6b* KO mice are phenotypically characterized by decreased numbers of mostly abnormal spermatids and spermatozoa (Roest et al., 1996). HR6B interacts with the E3s UBR2. The UBR2 also facilitates homologous recombination repair and maintenance of genomic integrity. *Ubr2* ^*−/−*^ male mice are sterile due to the deficiency in homologous pairing in meiosis I and apoptosis of spermatocytes in the pachytene stage (Kwon et al., 2003). UBR2 localizes to meiotic chromatin regions and regulates transcriptional silencing by ubiquitinating histone H2A. HR6B and UBR2 interact with the H2A substrate to enhance the transfer of ubiquitin from HR6B to H2A (An et al., 2010). RNF4 is a mammalian member of the E3s family targeting small ubiquitin-like modifiers (SUMO). The depletion of RNF4 dysregulates the formation of ubiquitin adduct at DSB sites and is associated with insistent histone H2A phosphorylation *in vitro*. The RNF4 accumulation at DSBs is involved in the regulation of protein turnover as well as exchange at these sites and is essential for the efficient repair of DSBs (Galanty et al., 2012). RNF4-defect mice show spermatocyte reduction and age-dependent impairment in spermatogenesis. Rad51, an enzyme required for DNA damage repair, is loaded onto damaged DNA sites by RNF4. Unlike UBR2, both homologous recombination and non-homologous end-joining repairs require RNF4 (Vyas et al., 2013). Histone H2B is monoubiquitinated at lysine 120 by E2s UbcH6 in the mammalian cells (Zhu et al., 2005). The mammalian E3s associated with H2B monoubiquitinating are RNF20 and RNF40. The *Rnf20* KO mice have a phenotype of small testes, loss of germ cells, and spermatocyte arrest in the meiotic I phase. The absence of RNF20 prevented chromatin relaxation. Due to this obstruction, the DNA repair factors are not proficiently recruited to program the DSB sites, which inhibit the repair of DSBs by meiotic recombination, ultimately leading to male infertility as a result of spermatocyte cell death (Xu et al., 2016). The abovementioned research studies show that HR6/RNF-dependent ubiquitination is involved in chromatin remodeling during spermatogenesis. During spermatogenesis, the histones are sequentially replaced by transition nucleoproteins and protamines. During sperm maturation, various histone variants have different degrees of monoubiquitination and polyubiquitination, which make the chromatin conformation loose and conducive to the removal and degradation of histones from chromosomes (Bao and Bedford, 2016). Although ubiquitin ligase RNF8 knockout mice can complete meiosis, a large number of histones are retained in abnormal sperm, the protamine fails to be loaded and normal mature sperm is absent, resulting in male mice infertility (Li et al., 2010; Lu et al., 2010). The clinical studies have found that the key infertility gene PIWI binds with RNF8 into a protein complex during spermatogenesis, and retains it in the cytoplasm, preventing the completion of histone ubiquitination, resulting in sperm maturation defects (Gou et al., 2017; Yan, 2017). DUB ataxin-3, a Josephin proteases family member, offsets the effects of RNF4 in response to the DSBs. In an early responses to DSBs, ataxin-3 counteracts RNF4 mediated MDC1 removal from chromatin. By retaining MDC1 at chromatin for enough time, ataxin-3 guarantees accurate differentiation of damage responses (Pfeiffer et al., 2017). Ataxin-3 and RNF4 coordinates their opposite activities to orchestrate robust MDC1-dependent signaling and DSB repair.

### Protein Ubiquitination and Deubiquitination Within the Post-Ejaculatory Sperm Maturation

The important post-ejaculatory sperm maturations such as sperm capacitation, acrosome reaction, penetrating zona pellucida, and elimination of sperm mitochondria after fertilization is also closely related to UPS. UBA1, an E1 responsible for ubiquitin activation, is necessary for fertilization. Despite seemingly normal motility and localization, the *uba-1* mutant *C. elegans* generates mature spermatozoa that are unable to fertilize to form zygotes (Kulkarni and Smith, 2008). This has also been verified in mammals. Porcine fertilization requires UBA1 to capitulate the sperm, induce acrosomal exocytosis, and penetrate the egg coat (Yin et al., 2012). NEDL2 are members of E3 ligases. NEDL2 antibody injection into porcine oocytes before IVF inhibits the formation of sperm DNA decondensation, reduces the number of male pronuclei, and leads to more zygotes without male pronucleus. The mice lacking E3 Herc4 exhibit mostly normal sperm morphologically but are defective in fertility and motility. (Rodriguez and Stewart, 2007). E3 ligase UBR7 is detected in mouse and boar testis and spermatozoa. Spermatozoa possess ubiquitin ligase activity in mammals. Despite the presence of the UBR7 protein in sperm acrosomes, it is not essential for sperm functions in fertilization (Zimmerman et al., 2014). Sperm capacitation is a necessary condition for fertilization, and where ubiquitination plays an important role by synergistic protein phosphorylation (Yi et al., 2012). During the sperm-zona pellucida interaction, UPS plays a dual role: 1) degrade proteins on the acrosome membrane after capacitation and trigger acrosome exocytosis; 2) promote the local degradation of the zona pellucida matrix and the formation of fertilization gaps (Saldívar-Hernández et al., 2015; Yi et al., 2015).

It is believed that USP2 plays a vital role in post-ejaculatory sperm maturation due to its exclusivity in late elongating spermatids (Lin et al., 2001). Despite normal sperm count and morphology, the *Usp2* ^−/−^ mice’s fertility was severely impaired. The intracytoplasmic sperm injection solved the problem, implying that the main defect is the sperm’s ability to fertilize the egg. The defect of *Usp2* ^*−/−*^ sperm lies in either binding or penetrating the ZP. The rapid loss of *Usp2* ^*−/−*^ sperm motility after transfer to nutrient-poor media implies defective abilities to generate chemical energies to convert chemical energies into mechanical forces (Bedard et al., 2011). The role of macrophage USP2 in modulating testicular function has been examined using a myeloid-specific gene knockout mouse model. The freshly isolated sperm were not affected by testicular macrophage USP2 in terms of morphology, viability, motility, or capacitation. Conversely, testicular macrophage USP2 promotes motility, hyperactivation, capacitation, and most exhilaratingly, the fertilizing capacity of frozen-thawed sperm *in vitro* (Hashimoto et al., 2021). USP8 is regarded as a biomarker for acrosomal biogenesis through the endocytic pathway since its significant role involves endosome sorting, vesicle trafficking, as well as endocytic vesicle maintenance (Mizuno et al., 2006; Berruti and Paiardi, 2015). In addition, USP8 contains a microtubule-interacting and transport domain that associates with the microtubule structure and the spermatid endosomal sorting complex required for transport-0 (Berruti et al., 2010). USP8 regulates both the endosomal pathway and the microtubule cytoskeleton that contribute to acrosome biogenesis.

### Sperm Selection Based on Ubiquitination and DUB Detection

The ideal approach for the sperm selection in IVF in the clinic should be non-invasive and cost-effective, by identifying high-quality sperm and producing better outcomes in terms of pregnancy and live birth rates. It is expected that the correlation between sperm ubiquitination levels and the sperm quality will be assessed by detecting sperm ubiquitination levels in infertile patients and whether it leads to better blastocysts. After spermatogenesis, the release of spermatozoa into the lumen occurs via various processes (Hess et al., 2008). Defective spermatozoa are ubiquitinated and can be eliminated by phagocytosis as a quality control mechanism before ejaculation (Pedrosa et al., 2020). Despite this mechanism, some defective spermatozoa labeled by extracellular/cell surface ubiquitination are carried into the seminal fluid (Muratori et al., 2007). Currently, during *in vitro* fertilization, the selection of sperm based on viability and morphology helped to avoid the participation of defective sperm marked by this ubiquitination in the fertilization process by detecting the level of sperm surface ubiquitination, which can lead to fertilization failure, pregnancy failure, and miscarriage (Sutovsky et al., 2001; Zarei-Kheirabadi et al., 2012).

The deletions in the region of the linked gene USP9Y are highly associated with infertility associated with oligospermia and azoospermia (Ferlin et al., 2007). Therefore, USP9Y has been considered a key gene for screening the Y chromosome for microdeletions in infertile or subfertile men (Simoni et al., 2004). However, as the study progressed, the fathers and brothers of USP9Y deficient patients were shown to have the same deficient USP9Y but were shown to have intact fertility. Inactivation of the USP9Y direct homolog in chimpanzees and bonobos (Tyler-Smith, 2008). The complete deletion of the USP9Y gene does not result in a spermatogenic defect and does not preclude natural conception. This is in line with the clinical case report in which complete deletions of the USP9Y gene were not associated with the development of spermatogenic defects (Luddi et al., 2009). The importance of USP9Y deletion alone for spermatogenesis is minimal, and the USP9Y gene is thought only to act as an efficiency-enhancing fine-tuner in human spermatogenesis. USP9Y is not vital for the production of normal sperms and fertility in humans. Therefore, USP9Y is not an appropriate indicator for sperm selection.

## Protein Ubiquitination and Deubiquitination in the Oocyte

The deficiencies in oocyte maturation directly contribute to ovulation disorders and infertility in females (Jose-Miller et al., 2007). The oocyte originates from the oogonia in the fetal stage. Oogonia differentiate into primary oocytes and begin meiotic prophase from nascent granulosa cells or the ovarian environment during embryonic development (Edson et al., 2009; Turathum et al., 2021). The oocytes are arrested at the meiotic prophase and form a tight complex with granulosa cells to create primordial follicles until puberty (Gosden and Lee, 2010; Coticchio et al., 2015). At this time, the nucleus of the oocyte is large and obvious and is called the germinal vesicle (GV). The first meiotic division resumes are triggered by follicle-stimulating hormone just before each ovulation (Kordowitzki et al., 2021). During this time, germinal vesicle breakdown (GVBD) occurs, and the antral follicles enter metaphase I (McGee and Hsueh, 2000). The chromosomes are condensed and the spindle migrates to the cortical region. The homologous chromosomes begin to separate. Upon ovulation, they are again arrested at metaphase II of meiosis II. If fertilized by sperm, these oocytes will resume and complete meiosis (Yang et al., 2019).

### Protein Ubiquitination and Deubiquitination in the Meiotic Process of Oogenesis

Because of the absence of transcriptional activity during oocyte meiosis, protein level regulation, especially PTM, is crucial for this process. Ubiquitination, as an important form of PTM, is known to play a key role in various cellular events during oocyte maturation and embryonic development, such as DNA damage response, chromosome condensation, and cytoskeleton organization (spindle morphology and segregation) (Table 2). Cyclin B1 is the core cyclin that regulates the meiotic progression of oocytes. The degradation of cyclin B1 largely affects the meiotic process of oocytes (Polański et al., 2012). The anaphase promoting complex (APC/C, an E3 ligase) is essential for cyclin B1 degradation in *S. cerevisiae*, *C. elegans*, and *Xenopus* (Masui, 2000; Bolte et al., 2002). As meiosis is arrested, cyclin B1 is ubiquitinated by APC/C for proteasome degradation. The low amounts of cyclin B1 leave CDK1 in a phosphorylation-inactive state. When the APC/C activity is suppressed, cyclin B1 accumulates and translocates to the nucleus to activate CDK1. This resulted in GVBD and resumed meiosis. Sister chromatid separation is an important process in the second meiosis of oocytes, which depends on the cleavage of mitotic adhesion proteins by separase. In mammals, separase is inhibited by binding to securin and CDK1/cyclin B. In mouse female meiosis II eggs, most of the separase is held in check by association with securin (Cohen-Fix et al., 1996). APC/C ubiquitinates the secruin for proteasome degradation and activates the separase to cleave the cohesin centromeres of sister chromatids. The activity of APC/C can be inhibited by the cytostatic factor Emi2. Only after the sharp increase in Ca^2+^ triggered by fertilization is Emi2 phosphorylated and inactive and resumes meiosis II.

**TABLE 2:** Ubiquitination and deubiquitination machinery and targeted substrates work to regulate oocyte development and function.

Enzyme	Substrate	Ub chain	Function of ubiquitination	Role in oocyte	Model	Reference
E2 enzyme						
UBE2S				First polar body emission	Mouse	Garnett et al. (2009)
Vih				Centriole elimination	*Drosophila*	Pimenta-Marques et al. (2016)
UEV1 & UBC13				Maternal membrane protein degradation	*C. elegans*	Sato et al. (2014)
UBC9				Spindle organization chromosome segregation	Mouse	Xu et al. (2020)
E3 ligase						
APC/C	Cyclin B1	Mono-ub K11-ub chains	Pro deg	Meiosis arrest	*C. elegans Xenopus* Mouse	Masui (2000), Bolte et al. (2002), Kirkpatrick et al. (2006), Szollosi et al. (1972)
APC/C	Polo	Unclear	Pro deg	Centriole elimination	*Drosophila*	Braun et al. (2021)
RFPL4	Cyclin B1	Unclear	Pro deg	Meiosis arrest	Mouse	Suzumori et al. (2003)
CRL4	PP2A PTEN	Poly-ub Poly-ub	Pro deg Pro deg	Meiosis promotion PI3K signaling activity	Mouse Mouse	Yu et al. (2015) Zhang et al. (2020)
MIB2	DLL3 (Notch ligand)	Unclear	quality of meiosis	AKT pathway activation	Mouse	Chen et al. (2021)
PRKN	RAB7	Unclear	Pro deg	Mitochondrial formation	Mouse	Koenig et al. (2014) Wang et al. (2019)
DUB						
UCHL1		Unclear		Polar body extrusion mulberry embryonic compaction	Boar Mouse	Luo et al. (2009) Mtango et al. (2012)
UCHL3		Unclear		Penetration into zona pellucida	Boar	Yi et al. (2007)

UBE2S, ubiquitin-conjugating enzyme E2 S; UEV1, ubiquitin-conjugating enzyme E2 variant 1; UBC13, ubiquitin-conjugating enzyme 13; UBC9, ubiquitin-conjugating enzyme 9; Pro deg, protein degradation; Mono-ub, monoubiquitin; Poly-ub, polyubiquitination; APC/C, anaphase promoting complex; RFPL4, ret finger protein-like 4; CRL4, cullin ring finger ubiquitin ligase 4; DLL3, delta Like canonical notch ligand 3 PRKN: parkin RBR; UCHL1, ubiquitin carboxy-terminal hydrolase L1; UCHL3, ubiquitin carboxy-terminal hydrolase L3;

The formation of polyubiquitin chains is one of the prerequisites for meiotic progression (Mogessie et al., 2018). APC/C attaches monoubiquitin to multiple lysine residues on cyclin B1 (Kirkpatrick et al., 2006). UBE2S, a member of E2s, can elongate the ubiquitin chains on the APC/C substrate cyclin B1 (Garnett et al., 2009). In oocytes resuming meiosis, K11, rather than K48 ubiquitin chains (the classical uniform of ubiquitin chains to target substrates for destruction by the proteasome) function as the main signal for degradation vital for first polar body emission (Pomerantz et al., 2012). Dimova and others demonstrate that the attachments of monoubiquitin to multiple lysine residues in cyclin B1 potentially generate a high density of ubiquitin that can also be recognized by the 26S proteasome. The formation of K11 ubiquitin chains may become necessary only when cyclin B1 does not possess sufficient lysine residues in *Xenopus* (Dimova et al., 2012). In mammals, RFPL4 (a member of E3s) associates with HR6A to attach ubiquitin to cyclin B1 and target it for proteasomal degradation (Suzumori et al., 2003).

Protein phosphatase 2A (PP2A) is another known meiosis regulator in oocytes. The E3s CRL4, together with its more than 90 substrate adapters DCAF1, binds to PP2A and targets it for proteasomal degradation and polyubiquitination to promote meiosis in mouse oocytes (Yu et al., 2015). CRL4 maintains the PI3K signaling pathway activity by targeting polyubiquitination and degradation of PTEN to stimulate meiosis resumption-coupled protein synthesis activation in mammalian oocytes (Zhang et al., 2020). The non-ATPase regulatory subunits (RPNs) are elements of 26S proteasomes in mammals. The activity of proteasomes is essential for the female reproductive process in *N. lugens.* The homologous sequences of Rpn in *N. lugens* are involved in the ovarian development and oocyte maturation (Wang et al., 2021). A multi-channel transmembrane protein, Gm364, binds and then anchors E3s MIB2 on cellular membranes. Then, MIB2 on membranes ubiquitinates and activates DLL3 which activates the cytoplasmic AKT pathway to control the occurrence and quality of the mouse oocyte meiosis (Chen et al., 2021).

UCHs are most relevant for the normal meiotic process during oogenesis among all the DUBs. This conclusion was confirmed by the application of UCH inhibition in different species, including murine (Mtango et al., 2012), bovine (Xu et al., 2020), porcine (Yi et al., 2007), and rhesus monkey (Mtango et al., 2012). UCHL1 localizes in the oocyte cortex and UCHL3 in the meiotic spindles. This localization is evolutionarily conserved in the mammalian oocytes, including humans, rhesus monkeys, mice, porcine, and cows (Mtango et al., 2014). UCHs suppression exerts its negative effects on meiotic spindle formation in oocyte maturation. UCHs may also influence spindle construction, spindle pole focusing, ubiquitin-mediated cyclin degradation, as well as chromosomal segregation in the course of metaphase–anaphase transitions (Huo et al., 2006; Susor et al., 2010; Tokumoto et al., 2020). The chromosomal organization is disturbed and the spindle length is altered among several properties of the abnormal meiotic spindle. Aneuploidy is witnessed in the oocyte and later the embryo may develop from such abnormalities (Mtango et al., 2012).

UCHL1 may regulate microfilament-containing actin and myosin during oocyte meiotic polar body extrusion (Mtango et al., 2014). Apart from the dysregulated microtubule and chromosomal organization within the meiotic spindles, the suppression of spindle-related UCHL3 alters spindle pole-to-pole distance as well as spindle pole width. From metaphase to anaphase, the distance between the spindle poles remains constant. Among the possible substrate proteins of UCHL3, separase/securin is an enzyme of the meiotic spindle (Yanagida, 2005). These suggest an important role for UCHs in oogenesis.

### Regulation of Ubiquitination in the Elimination of Oocyte Centrioles

The elimination of oocyte centrioles takes place during the meiosis stage in most animals, including humans (Hertig and Adams, 1967; Szollosi et al., 1972; Gruss, 2018). The fertilized zygote depends on the sperm to provide the paternal centriole. The embryo is prevented from developing parthenogenetically. The embryo centrosome number is balanced and interference with meiotic and mitotic divisions is avoided after fertilization (Manandhar et al., 2005). It has been recently demonstrated that centrioles are eliminated from the mammalian oocyte at the late stage of oogenesis (Pimenta-Marques et al., 2016). This has been studied in detail in *Drosophila*. Only when the centrosomes within the *Drosophila* syncytial cysts migrate to the oocyte the centrioles are able to degenerate. As APC/C activity is suppressed, the centrioles migrate and the oocyte is maintained in the pre-meiotic arrest state. APC/C then turns back on to degrade Polo to eliminate the centrioles from the oocyte (Pimenta-Marques et al., 2016). The APC/C and its specific E2 Vih (mammal homologs, Ube2c) are essential for this migration see Table 2. The mutations in Vih cause abnormalities in the oogenesis of *Drosophila*. This may be due to the disruption of the negative regulation of APC/C by the Vih mutation, thus leading to excessive degradation of Polo and inability to migrate centrioles first and oocyte failure to occur normally (Braun et al., 2021). Given the preserved functions of the centrioles and ubiquitin in mammals, these findings may apply to other species. The change in APC/C activity does not exactly match the above, which may be due to the fact that APC/C is very carefully regulated to affect polo kinase and has not yet reached the threshold to affect cell cycle proteins. Of course, this is expected to be verified in further studies on different species.

### Regulation of Ubiquitination in Mitochondrial Biology

Dysfunction in one or more parts of mitochondrial biology has been reported to decline oocyte quality. For instance, reduced intracellular ATP content reduced mtDNA numbers and accumulation of mtDNA point mutants, and altered membrane potential (May-Panloup et al., 2016). A study recently reported that RAB7 is more and more ubiquitinated by PRKN (an E3 molecule) and degrades through the proteasome as women age. The lower level of RAB7 leads to a defective mitochondrial formation and accumulation of damaged mitochondria, which decreases the oocyte quality (Jin et al., 2021). The ways in which protein ubiquitination and deubiquitination participate in the maintenance of functional mitochondria in oocytes is also worth investigating.

### Ubiquitination and Deubiquitination Regulate Communication Between Oocytes and Granulosa Cells

The ovarian somatic cells, especially granulosa cells, perform an indispensable function in promoting female germ cell development, including providing a niche for oocyte survival, nourishment, meiotic arrest, and resumption (Gosden and Lee, 2010; Saitou and Hayashi, 2021). In follicular granulosa cells, the ubiquitination-proteasome system plays a key role in the regulation of cumulus extracellular matrix deposition and steroid productions via cumulus cell expansion, indicating that this system is vital for follicular and oocyte development.

The downregulated content of SUMO-1 in mouse granulosa cells is related to the high luteinizing hormone (LH) level. LH is the hormone most correlated with the resume of oocyte maturation as well as the signal for ovarian progesterone production. The SUMO-1 interacts with the progesterone receptor to affect the ovulatory process. Progesterone on the other hand inhibits the expression of SUMO-1 (Shao et al., 2004). Inhibiting SUMO-1 causes disordered localization of γ-tubulin and disrupted kinetochore-microtubule attachment at metaphase I during mouse oogenesis. The incorrect assembly of spindle microtubules and less-condensed chromosomes decreased the rates of GVBD and oocyte maturation (Yuan et al., 2014). The NEDL2 is a member of the NEDD4 subfamily of E3 ligases. The level of NEDL2 in the cumulus cells of oocytes experiencing *in vitro* maturation increased 10 times than the porcine oocyte in the LH and FSH free medium. This indicates that NEDL2 may play an important role in the granulosa cells during oocyte maturation (Mao et al., 2020).

USP9X and Afadin co-localize and are simultaneously downregulated in the granulosa cells during oocyte maturation. Afadin is a component responsible for cell-cell adhesions and a potential substrate of USP9X. USP9X may deubiquitinate and stabilize Afadin to participate in the process (Sato et al., 2014). However, it is a pity that the authors stopped here without further confirmation. The inhibition of UCHL1 results in elevations of K63-associated polyubiquitin chain abundance in bovine oocytes, and cortical granules migration toward the cortex during oocyte maturation is impaired (Susor et al., 2010). Moreover, the ubiquitination-proteasome system regulates cumulus extracellular matrix deposition and steroid production in the course of cumulus expansion in domestic porcine (Nagyova et al., 2012).

## Protein Ubiquitination and Deubiquitination in Early Embryonic Development

### Ubiquitination in Devastating Paternal Mitochondria

All mammals including humans inherit maternal mitochondria, although the sperms contribute about one hundred mitochondria to the zygote. This requires an accurate mechanism of identifying and devastating the paternal origin of the mitochondria. Ubiquitin works as a recycling marker to tag sperm mitochondria inside fertilized bovine and monkey eggs. The death sentence is imprinted during spermatogenesis and executed when the mitochondria of the sperm are contacted by the cytoplasmic destruction machine of the egg (Sutovsky et al., 1999). Autophagy, as well as the ubiquitin-proteasome system, is involved in mitochondrial autophagy in mammalian sperm after fertilization (Song et al., 2016). SQSTM1, an autophagy receptor that binds to ubiquitin, may identify ubiquitinated mitochondrial proteins and associate with ubiquitin-like modifications, including GABARAP and/or LC3. The ubiquitinated mitochondrial proteins are extracted to form aggresomes transported to autophagophores. VCP, the protein dislocates, extracts, and presents the ubiquitinated mitochondrial membrane proteins to 26S proteasomes. Ubiquitination directs sperm mitochondrial recognition and processing during preimplantation embryo development, thereby preventing the potentially deleterious effects of heterogeneity.

### Protein Ubiquitination and Deubiquitination in Maternal-to-Zygotic Transition

Maternal-to-zygotic transition (MZT) is a turning point for embryo development, where the embryos escape maternal genome control for the first time and use the zygotic genome for transcription. MZT consists of two key processes: the clearance of proteins from the eggs and the activation of the zygotic genome (ZGA). The proteomic analysis revealed that ubiquitin-related proteins and DUBs are highly enriched in mammalian zygotic (Mtango and Latham, 2007; Wang et al., 2010), which indicates that protein ubiquitination and deubiquitination are essential for mouse MZT. Ubiquitination mediated by the E3s rnf114 targeting the degradation of TAB1 may be required for NF-κB pathway activation during mouse MZT. This is directly related to maternal clearance with early embryonic development (Yang et al., 2017). Sperm and egg fusions induce transient K63-linked polyubiquitylation, resulting in ubiquitylated proteins accumulating on endosomes. The E2 enzymes, UEV1 and UBC13 are responsible for sorting the maternal membrane proteins to lysosomes for degradation in *C. elegans* by mediating the K63-linked polyubiquitylation (Sato et al., 2014).

The initiation of transcription by the syngeneic genome after the removal of maternal mRNAs and proteins is termed ZGA. ZGA occurs in a precisely timed controlled manner with three major classes of activation model hypotheses: 1) karyoplasmic ratio (N/C): a threshold ratio of nuclear components to cytoplasmic volume mitigates transcriptional repression (Schulz and Harrison, 2019); 2) maternal clock model: maternal deposition of activating or repressing transcription factors may determine the timing of gene expression; and 3) re-establishment of chromatin state allowing transcription of the syncytial genome (Jukam et al., 2017). PTM is essential for ZGA and is associated with the regulation of N/C, transcriptional repression and activation, regulation of chromatin state remodeling, and histone modifications. The inactivation of proteasomes results in the arrest of most zygotes (Shin et al., 2010). This implied that protein ubiquitination has critical effects on epigenetic reprogramming, such as post-fertilization histone modifications. It was shown that monoubiquitination of histone H2A (H2Aub1) and chromatin compaction jointly regulated the transcriptional repression in embryonic stem cells. H2B1ub is expressed at the late 1-cell to the blastocyst stage (Plusa et al., 2008). SUMOylation mediated by the E2 enzyme UBC9 affects meiosis and preimplantation development by regulating spindle organization and chromosome segregation (Xu et al., 2020). The deletion of *ubc9* leads to embryonic lethality due to defective chromosome segregation and lack of integrity in the nucleus of mouse embryos and inhibition of *ubc9* during the GV phase leads to disruption of spindle structure, thereby preventing meiotic maturation. Adding exogenous UBC9 to the oocyte maturation medium decreased SUMO-1 levels, thereby inhibiting PB1 efflux and reducing oocyte maturation. During embryonic development, overexpression of the E3 ligase PIASy allows abnormal chromosome segregation and impaired syncytial transcription leading to embryonic arrest at the 2-cell stage. The overexpression of PIASy in mouse fertilized eggs inhibits ZGA and impairs early embryonic development (Higuchi et al., 2019).

## Concluding Remarks and Future Perspectives

Here, we elucidated several ubiquitinated and deubiquitination modifications in cells and highlighted recent advances in their use in mammalian reproduction. Any error during protein ubiquitination and deubiquitination may result in the inability to intercept specific proteins for degradation or maintaining the ubiquitin pool. Dissecting the diverse mechanisms underlying ubiquitin and DUBs in vital cellular functions allowed us to comprehend how protein ubiquitination and deubiquitination drove and regulated the various reproductive processes. The process of histone ubiquitination which dynamically regulates nucleosome stability and chromatin dynamics through ubiquitination and deubiquitination during oocyte maturation and preimplantation could be a hot topic for future research.

Although there is a correlation between the degree of ubiquitination and sperm quality, ubiquitination in the epididymis removes defective sperm, and the reorganization of ubiquitinated proteins onto the sperm surface after capacitation is part of the interaction of the sperm oocyte with the zona pellucida. These proteins may be eliminated by the protease system of the oocyte after fertilization. Therefore, it cannot be used as a candidate for clinical sperm quality. As the function and mechanisms of ubiquitin and DUBs continue to be investigated, through future testing of key deubiquitinating enzymes during gametogenesis and the level of ubiquitination at crucial time points, we have the opportunity to test whether the abnormalities in these genes can serve as the basis for an accurate molecular diagnosis of clinical infertility in couples. Targeting the revitalization of ubiquitin ligases and key deubiquitinating enzymes promotes better ovulation, selection of high-quality sperm, and even better fertilization rates, and blastocyst quality or even improved live birth rates. An opportunity is presented to test whether the abnormalities in these genes may serve as a basis for precise molecular diagnosis of clinical infertility in couples and may one day provide specific therapeutic approaches for IVF. However, with the dynamic change in protein ubiquitination and deubiquitination during gametogenesis, the timing of ubiquitination interventions in ART/IVF should be used with particular care. It will contribute to a comprehensive understanding of early human embryonic development mechanisms and provide clues to solve various problems in clinical reproductive medicine, including infertility and early miscarriage.

On the other hand, another potential clinical application for DUBs is the potential target for birth control development. Since DUBs are enzymes, they are theoretically suitable for high-throughput analysis for screening drug candidate inhibitors. The targeted enzymes are somewhat germ cell-specific and may be less likely to be toxic to other cell types. We expect that a further research into protein ubiquitination and deubiquitination will pay off with basic science that can decipher intracellular homeostasis and improved therapeutic approaches that will benefit all of humanity by providing clues to solve various problems in clinical reproductive medicine.
